# 3D Printed Laminated CaCO_3_-Nanocellulose Films as Controlled-Release 5-Fluorouracil

**DOI:** 10.3390/polym12040986

**Published:** 2020-04-23

**Authors:** Denesh Mohan, Nur Fatin Khairullah, Yan Ping How, Mohd Shaiful Sajab, Hatika Kaco

**Affiliations:** 1 Research Center for Sustainable Process Technology (CESPRO), Faculty of Engineering and Built Environment, Universiti Kebangsaan Malaysia, Bangi 43600, Selangor, Malaysia; denesh.mohan@gmail.com (D.M.); p92985@siswa.ukm.edu.my (N.F.K.); cerisedawn0417@gmail.com (Y.P.H.); 2 Department of Chemical and Process Engineering, Faculty of Engineering and Built Environment, Universiti Kebangsaan Malaysia, Bangi 43600, Selangor, Malaysia; 3 Kolej GENIUS Insan, Universiti Sains Islam Malaysia, Bandar Baru Nilai, Nilai 71800, Negeri Sembilan, Malaysia; hatikakaco@usim.edu.my

**Keywords:** 3D printing, 5-FU, drug delivery, liquid deposition modelling, nanocellulose

## Abstract

Drug delivery constitutes the formulations, technologies, and systems for the transport of pharmaceutical compounds to specific areas in the body to exert safe therapeutic effects. The main criteria for selecting the correct medium for drug delivery are the quantity of the drug being carried and the amount of time required to release the drug. Hence, this research aimed to improve the aforementioned criteria by synthesizing a medium based on calcium carbonate-nanocellulose composite and evaluating its efficiency as a medium for drug delivery. Specifically, the efficiency was assessed in terms of the rates of uptake and release of 5-fluorouracil. Through the evaluation of the morphological and chemical properties of the synthesized composite, the established 3D printing profiles of nanocellulose and CaCO_3_ took place following the layer-by-layer films. The 3D printed double laminated CaCO_3_-nanocellulose managed to release the 5-fluorouracil as an effective single composition and in a time-controlled manner.

## 1. Introduction

The concept of drug delivery systems revolves around the routes of administration with the main ones being oral, topical and respiratory, and dosage. Meanwhile, drug delivery technologies transform the profiles, absorption, distribution, and elimination of drugs to improve product safety, and the health compliance of the patient [1]. Meanwhile, drug release also refers to the methods of the deployment of a drug, for example, through degradation, swelling, and affinity-based mechanisms [2]. In previous and current practices, proteins and phospholipids are the primary drug delivery media [3]. The benefits of proteins include their permeability and hydrophilicity. However, their major drawbacks are the high production cost, low bioavailability, easy degradability when transported in drug delivery systems, and toxicity, which can adversely affect patient health [4,5]. Meanwhile, phospholipids are a viable option because of their biocompatibility and amphiphilicity. These unique characteristics have enabled phospholipids to be utilized in numerous drug delivery systems. Nevertheless, similar to proteins, it is difficult to control the purity of phospholipids during production [6]. Additionally, their instability causes them to be metabolized into lysophospholipids during use or storage [3]. Hence, many researchers have attempted to overcome such problems.

One of the drugs that have been commercially used for chemotherapy in cancer treatment is 5-fluorouracil (5-FU) [7]. 5-FU shows promising signs in treating various types of cancers, such as breast cancer, stomach cancer, head and neck cancer and colon cancer [8,9]. Being a chemotherapeutic agent that prevents pyrimidine metabolism and DNA synthesis, fluorouracil, and its metabolites involves various mechanisms of action [10]. Specifically, fluorouracil activates its metabolite, 5-fluoroxyuridine monophosphate, in vivo to replace its uracil, which is one of the nucleotide bases of ribonucleic acid (RNA) [11]. Accordingly, fluorouracil can combine with the target RNA, inhibit the processing of the RNA, and, in turn, prevent the division of infected or mutated cells. The second mechanism involves the activation of another metabolite—5-5fluoro-2′-deoxyuridine-5′-O-monophosphate—which then inhibits thymidylate synthase, resulting in the depletion of triphosphate thymidine (one of the four types of triphosphate nucleotides needed for the in vivo synthesis of deoxyribonucleic acid (DNA)). Meanwhile, the other metabolites of fluorouracil affect the structure, processing, and functions of both DNA and RNA [12]. In spite of that, the usage of 5-FU in treatment comes with side effects to the patients such as diarrhea, nausea, vomiting, mouth sores, poor appetite, photosensitivity, metallic taste, neutropenia, thrombocytopenia and cognitive impairment [13,14]. Thus, the loading and controlled release of the drug has become ever more essential for the drug delivery system. The main benefit of controlled release is the safety and the reduction in the negative effects of the rapid initial release of the drug into the body [15].

Nanocellulose has been used as a drug carrier in various forms, such as hydrogels, and biopolymer composite, and has been found to have the capability of a controlled drug release [16]. Whereas the research on 3D printed nanocellulose is still at the beginning stage, the studies on nanocellulose have expanded vastly. The printing ink preparation, condition, solidification method, and mechanical properties of the printed nanocellulose product are being studied intensively [17,18,19,20]. For example, a low concentration of cellulose nanofibrils (CNF) can be utilized as a 3D printable ink due to its characteristics of shear-thinning and high mechanical properties gained from the ability to get entangled [21]. The feature of shear-thinning at an increased shear rate when passing through the nozzle is highly sought in liquid deposition modeling (LDM)-based 3D printing [22]. The zero-order controlled release drug profile over 24 h was able to be obtained using 3D printed hydroxypropyl-ethyl cellulose tablets [23]. Furthermore, a sustained-release drug profile from a CNF hydrogel was modulated using different geometries of 3D-printed polylactic acid (PLA) capsules to attain the desired controlled-release profile [24].

In the current study, a laminated calcium carbonate-nanocellulose film—which had the potential to transcend the controlled drug release—was synthesized. For starters, calcium carbonate (CaCO_3_), or limestone, is an abundant natural inorganic compound widely used in biomedicine owing to its biocompatibility, pH-sensitivity, and slow degradation [25]. CaCO_3_ was chosen to be a component of our composite (i.e., drug delivery medium) because its pH sensitivity enabled a target agent to adopt a unique feature for the benefit of the drug delivery system [26]. Meanwhile, the basis for the use of cellulose in the current study was that it is the most abundant biopolymer. Furthermore, since cellulose is a renewable polymer, it has many applications in the biomedical and pharmaceutical fields. Besides that, its biocompatibility, biodegradability, cost-effectiveness, minimal risk of ecological toxicity, and reasonably low toxicity to human and animal cells make it a highly suitable drug delivery medium [27]. Nanocellulose synthesized from the process of fibrillation of cellulose was used as it has a high surface area to volume to increase the calcium carbonate and drug binding at the surface. CaCO_3_ was chosen to be coupled with CNF due to its low toxicity and capability of the targeted delivery system, while CNF was used as it is a promising material for controlled drug delivery owing to its biocompatibility, mechanical properties and renewable nature.

Hence, the study focused on the usage of 3D printed CaCO_3_-nanocellulose models to attain a controlled release of the loaded therapeutic drug. CNF was isolated and defibrillated from the oil palm biomass of empty fruit bunch (EFB) fibres, and an intensive research was done on the printing profile to print circular-shaped adsorbent. CaCO_3_ was synthesized from the precipitation reaction of calcium chloride and sodium carbonate. Similar to CNF, an intensive research was done on CaCO_3_ printing as well. Furthermore, different types of CaCO_3_ and CNF models were 3D printed to study on the controlled released of 5-FU: CNF mixture with CaCO_3_, CNF laminated with CaCO_3_, and CNF double-laminated with CaCO_3_.

## 2. Materials and Methods

### 2.1. Materials

Oil palm EFB fibers were purchased from Szetech Engineering Sdn Bhd (Selangor, Malaysia) at desired sizes of 106 to 500 μm. The isolation of cellulose was done using formic acid, 90%, sodium hydroxide, hydrogen peroxide and H_2_O_2_ (Merck, Darmstadt, Germany) and was catalyzed by Fe(II) prepared from Fe_2_SO_4_ (Merck). The preparation of CaCO_3_ nanoparticles was done using calcium chloride, CaCl_2_, and sodium carbonate, Na_2_CO_3_ (Merck). The stock solution of 5-fluorouracil (Merck) was prepared at 100 ppm in sodium acetate solution (Merck) while the 10× concentrated phosphate-buffered saline, PBS (Merck), was diluted to a phosphate buffer and sodium chloride concentration of 0.01 and 0.154 M, respectively.

### 2.2. Preparation of CNF and CaCO_3_–CNF Laminated Films

The isolation of cellulose from oil palm EFB fibres was performed, referring to the protocol from the previous study [28,29,30]. Oil palm EFB was reacted with formic acid (90% *w/v*) with a ratio of 1:30 at 95 °C for 2 h. The experiments were conducted in three-necked flat-bottom flasks equipped with a condenser on a digital hotplate magnetic stirrer (MSH-20D, Daihan Scientific, Gangwon-do, Korea) at 800 rpm. The separation of pulp and supernatant was separated using a vacuum filter (MVP 10, IKA, Staufen, Germany). The pulp was reacted with NaOH (2 wt %) and H_2_O_2_ (2 wt %) as the bleaching process for the removal of lignin and hemicellulose. Catalytic oxidation was done with 10 mg/L with H_2_O_2_ (2% *w/v*) at 90 °C for 24 h to enhance the purification of cellulose fraction. Further washing was done to the pulp obtained to remove chemical residues and kept at 4 °C for further use. The purity of the cellulose that was fractionated was studied according to the National Renewable Energy Laboratory (NREL) standard. To yield CNF by the homogenization process, 0.7 wt % of cellulose solution was fibrillated using a high-speed blender at 37,000 rpm (Vitamix 5200, Vitamix, OH, USA) for 30 min while also keeping the temperature below 70 °C throughout the process to avoid hydrolysis of cellulose. The fibrillated cellulose was stored at 4 °C in a refrigerator.

The preparation of CaCO_3_ was conducted by mixing a 1:1 molar ratio of CaCl_2_ and Na_2_CO_3_ in deionized water at 200 rpm for 2 h. Sedimented CaCO_3_ was washed, centrifuged, and dried at 50 °C until a constant weight was achieved. While the preparation of CNF and CaCO_3_–CNF laminated films were cast by vacuum filtration technique. Briefly, the prepared CNF and CaCO_3_ suspensions (0.1 g dry weight basis) were deposited through a nylon membrane filter (47-mm diameter, 0.45-µm pore size) in a single composition and a layer by layer deposition of CaCO_3_–CNF films. The casted films were dried using a dehydrator overnight and kept in a desiccator until further use (see Figure S1 in Supplementary Materials).

### 2.3. Liquid Deposition Modelling

Discov3ry paste extruder from Structur3D Printing, Kitchener, Canada was used to integrate with the Ultimaker 2+ 3D printer (Ultimaker, Utrecht, Netherlands) to enable us to print liquid paste. A computer aided design (CAD) model, which was modeled in the .stl file was converted to a G-code file through slicer software (Ultimaker Cura 4.5, Geldermalsen, Netherlands) to be read by the printer for the extrusion and movement of the print head according to the desired model. The printer’s glass build plate was cleaned with isopropanol before printing so that the printed material can be removed smoothly after printing for further study. The nozzle of 1.21 mm internal diameter was used throughout the study.

A circular model film of 40 mm diameter was designed to carry out the total uptake and release of the 5-FU process. Before printing, the CNF fibrillated was centrifuged to obtain 7 wt %, and CaCO_3_ was prepared to 7 wt % using deionized water to have a paste-like printable solution. The solution was then fed in the syringe and centrifuged at 1500 rpm for 10 min to remove the gas bubbles from the solution for smooth extrusion. The desired printing profile was studied using a circle (20 mm radius x 1.21 cm height) model to optimize the CNF and CaCO_3_ printing profiles.

For the CNF mixture with CaCO_3_ (CaCO_3_–CNF–M) in the adsorption study, CaCO_3_ and CNF were mixed in the ratio of 1:1 before printing. While CNF laminated with CaCO_3_ (CaCO_3_–CNF–L) and CNF double laminated with CaCO_3_ (CaCO_3_–CNF–DL) on adsorption of 5-FU study, CNF is printed first, and the solution was changed to CaCO_3_ and start printing on the subsequent layers. Each parameter was performed in three replicates and the printed samples were dried in at 80 °C for 8 h before the uptake and release of 5-FU study. The 3D printed films were dried using a dehydrator overnight and kept in a desiccator until further use.

### 2.4. Total Uptake and Release of 5-FU

In this experiment, the total uptake study was performed in three replicates using the adsorption phenomena by mixing the printed samples in 30 mL of 5-FU (1–10 ppm) for 24 h at 200 rpm at room temperature. The final solution of 5-FU was withdrawn and analyzed by UV-Vis spectrophotometry. The total uptake amount of 5-FU at final equilibrium (*q*_e_) can be calculated using the following equation:

(1) $$q_{e}=\frac{\left(C_{o}-C_{e}\right)V}{m}$$

where *C*_0_ and *C*_e_ are the initial and equilibrium concentrations of the 5-FU (mg/L), respectively, *V* is the volume of the solution (L), and *m* is the mass of the adsorbent (g) [31].

Consecutively, the mixture of CaCO_3_ and CNF with 5-FU was centrifuged at 11,000 rpm for 20 min and dried at 50 °C to constant weight. The release of 5-FU has been carried out in PBS solution for 24 h at 200 rpm at room temperature. The amount of 5-FU release from CaCO_3_ and CNF was calculated using the following equation.

(2) $$5\text{-}\mathrm{FU}\;\mathrm{release}\;\left(\%\right)=\frac{5\text{-}\mathrm{FU}\;\mathrm{concentration}\;\mathrm{released}\;(\mathrm{mg}/L)}{5\text{-}\mathrm{FU}\;\mathrm{concentration}\;\mathrm{adsorbed}\left(\mathrm{mg}/L\right)}\times 100\%$$

### 2.5. Characterization

The micrograph images of CNF and CaCO_3_ was observed through a field emission scanning electron microscope (FESEM) (Merlin Compact, Zeiss Pvt. Ltd., Oberkochen, Germany). The crystalline region of the samples was performed using an X-ray diffractometer (XRD) (Bruker D8 Advance, Bruker, Billerica, MA, USA). The functional group spectrum was done using attenuated total reflectance Fourier transform infrared spectroscopy, ATR-FTIR (ALPHA Fourier transform infrared (FTIR) Spectrometer, Bruker, Billerica, MA, USA) at a resolution of 1 cm^−1^ in the range of 4000 to 650 cm^−1^. The mechanical properties of the 3D printed samples (standard tensile specimen according to the ASTM D638 Type IV in Figure S2) were evaluated using the Instron^®^ Electromechanical Universal Testing Systems 3300 Series at 1 mm/min with a load cell of 100 N. While the concentration of 5-FU was monitored using UV-Vis spectrophotometry (Single beam UV spectrophotometer SP-UV 300SRB, Spectrum Instruments GmbH, Überlingen, Germany) and calibrated at the maximum wavenumber, λ_max_ of 266 nm, which followed to the linearity of the Lambert-Beer law principal.

### 2.6. Analysis of Variance

The results are expressed in the form of mean ± standard deviation (SD). All the data reported were done based on the mean of three replicates (*n* = 3). The analysis was done with Minitab ^®^(statistical software release 18, Minitab, State College, PA, USA). One-way analysis of variance (ANOVA) followed by Tukey’s Test with significance level criteria of *p* < 0.05 was performed to determine whether or not the different drug release models CaCO_3_, CNF, CaCO_3_–CNF–M, CaCO_3_–CNF–L, CaCO_3_–CNF–DL are all equal.

## 3. Results and Discussion

### 3.1. Characterization

**Figure 1:**
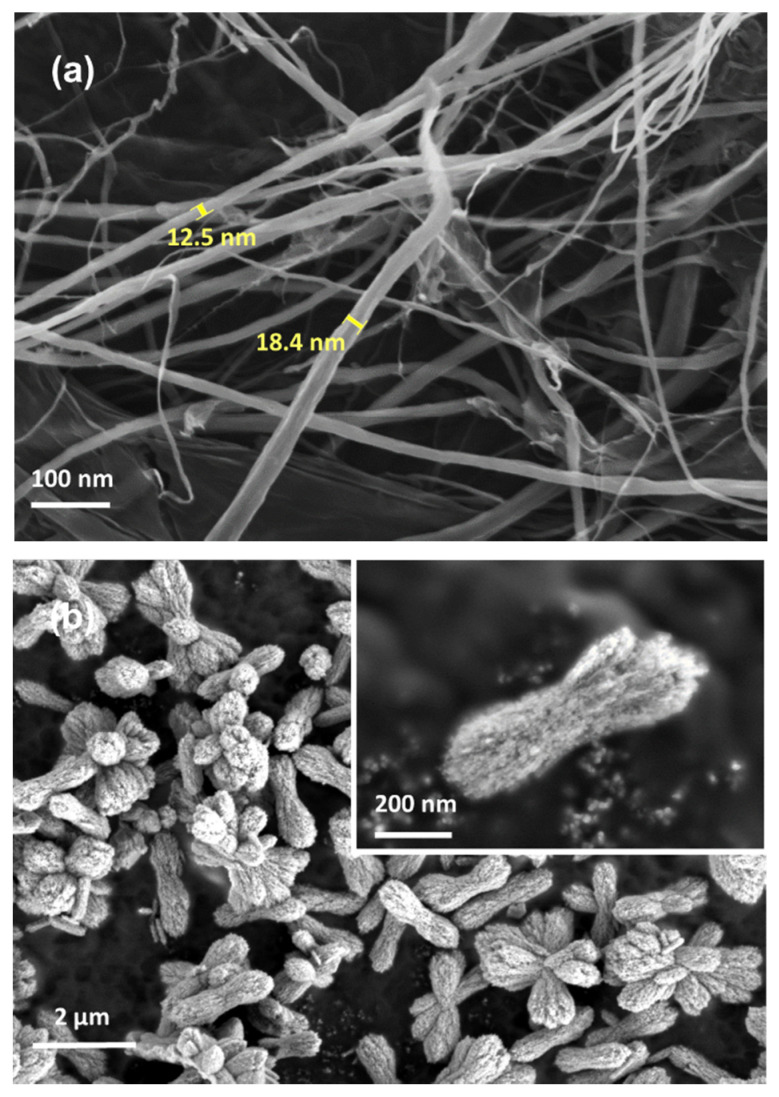
Micrograph images of (**a**) isolated and defibrillated cellulose and (**b**) synthesized CaCO_3_ by field emission scanning electron microscope (FESEM) analysis (inset shows the magnified image).

As observed in Figure 1a, the diameter of the fibre was less than 20 nm because of the mechanical treatment, leading to scattered and web-like matrix structures [28]. The morphological images of the cellulose nanofibril network indicated that they became individually separated owing to the removal of hemicellulose and lignin after the chemical treatment and the fibrillation process, causing the disintegration [30]. The synthesized CaCO_3_ crystals exhibited multiple porous structures with high surface area as desired for the adsorption process through the precipitation reaction of sodium carbonate and calcium chloride, as shown in Figure 1b. Referring to previous studies, the CaCO_3_ shape obtained was vaterite due to its irregular shape and flower-like particle, which benefited this study since the shape had good prospects in being drug delivery carriers [32,33].

**Figure 2:**
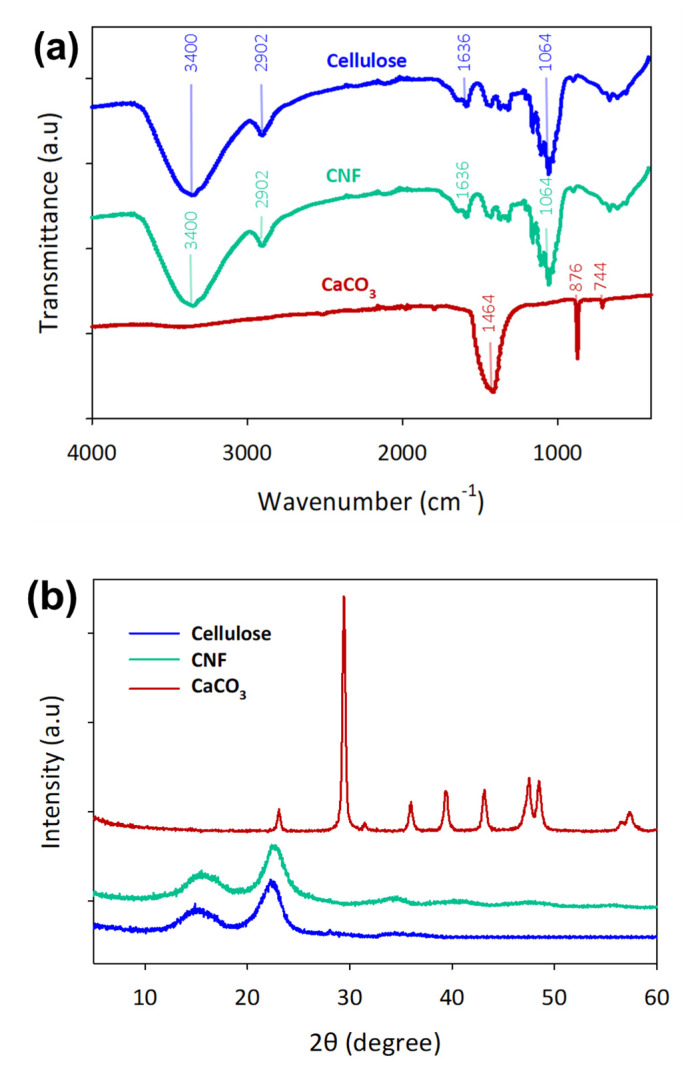
Characterization of isolated cellulose, cellulose nanofibrils (CNF) and CaCO_3_ on (**a**) Fourier transform infrared (FTIR) spectroscopy and (**b**) the X-ray diffractometry (XRD) spectrum.

Figure 2a shows that the FTIR absorption peaks at 3400, 2902, and 1058 cm^−1^ are attributed to the aliphatic C–H, C–O stretching vibrations, respectively, and they are present in cellulose and CNF [29]. No bands were observed at 1733, 1258, and 1044 cm^−1^ owing to the removal of lignin and hemicellulose from the oil palm EFB fibres [34]. FTIR spectra obtained for the CaCO_3_ samples demonstrated a broad absorption peak of CO_3_^2−^ at 1464, 876, 744 cm^−1^, which were the vaterite peaks present in calcium carbonate [35]. The XRD pattern of cellulose 1 was well matched around 14.5, 16.5 and 22.5°, attributing to the planes of (110, $1\bar{1}0$, 200) for both cellulose and CNF [36]. CNF crystallinity increased to 76.02% from 63.05% of cellulose due to the defibrillation process. These results show that the calcium carbonate synthesized was highly crystalline with a crystallinity index of 84.9°, and the peaks were well matched with the diffraction peaks of vaterite CaCO_3_ compared to the previous studies [37,38].

### 3.2. Total Uptake and Release of 5-FU Using CNF and CaCO_3_ Casted Films

**Figure 3:**
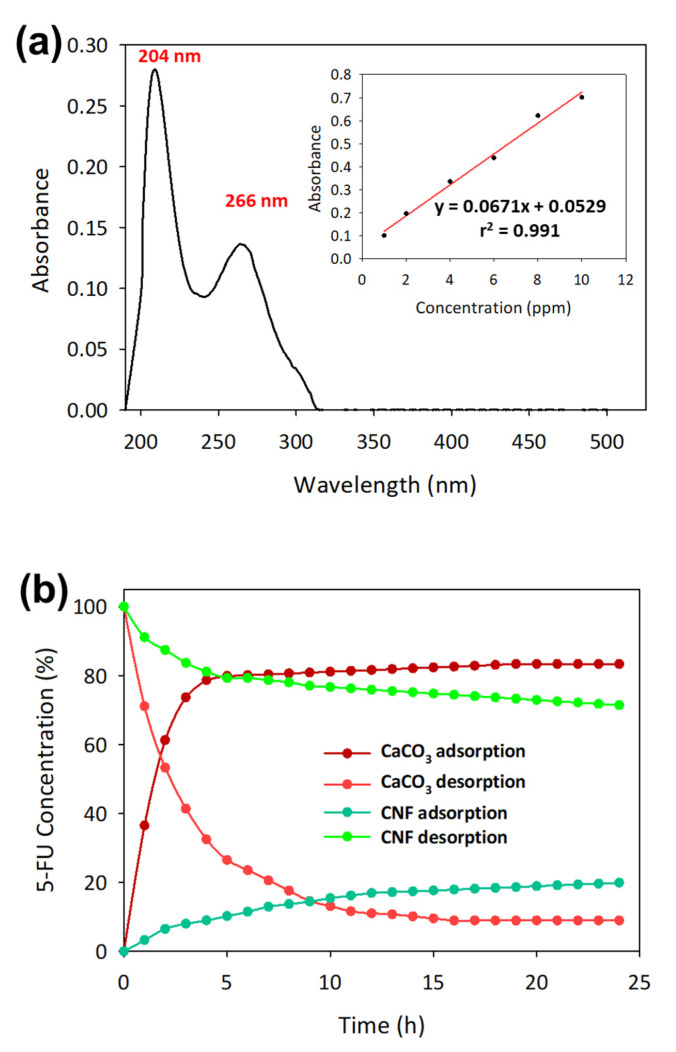
(**a**) Spectrum of 5-fluorouracil (5-FU) using a spectrophotometer (inset shows linear calibration curve according to the Beer-Lambert law) and (**b**) the adsorption-desorption kinetics of 5-FU using casted films of CNF and CaCO_3_. The data are presented as mean (*n* = 3).

The absorption spectrum of 5-FU was recorded between 190 and 500 nm. The optimal wavelength of 5-FU absorption using UV was at 204 and 266 nm by measuring the standard 5-FU solution in a sodium acetate buffer solution for 5-FU concentrations of 1–10 ppm [39]. We chose 266 nm to be the most optimum wavelength because the absorption spectra near 200 nm were absorbed by atmospheric oxygen that could affect the accuracy of the measured absorbance [40]. 5-FU solution at concentrations of 1, 2, 4, 6, 8, and 10 ppm was used to identify the standard linear curve. Based on Figure 3a, the graph equation shows a high regression value, r^2^ of 0.991, indicating a good correlation between the experimental and theoretical values.

As shown in Table 1, the 5-FU uptake and release process was carried out using CaCO_3_, CNF and CaCO_3_–CNF casted films. CaCO_3_ showed a higher uptake and release of 5-FU owing to the high surface area caused by the vaterite crystalline polymorph [41]. The highest uptake of 5-FU was up to 83.19% for the 5-FU initial concentration of 6 ppm and the release percentage was recorded at 91.20%. At the same initial concentration of 5-FU, CNF film showed very low uptake at 19.86% and release at 28.59%. The adsorption capacity of 5-FU on the CNF was in the range 0.05–0.65 mg/g and was comparable with a previous study [28]. This proves that nanocellulose is not suitable to be loaded with 5-FU but adequate to be used as a drug carrier for controlled release [42]. However, laminated CaCO_3_–CNF film shows a significant reduction in 0.03 mg/g on the total amount of 5-FU in comparison with CaCO_3_ film. While the lamination of CNF on the CaCO_3_, preventing the total release of 5-FU. Consequently, 6 ppm was chosen to be the ideal concentration to carry out the adsorption and desorption process using a different type of CaCO_3_, CNF and CaCO_3_–CNF casted films, which showed the highest release for CaCO_3_.

Referring to Figure 3b, the adsorption-desorption kinetics were compared between CaCO_3_ and CNF, and the time taken for 80% of the 6 ppm 5-FU to be adsorbed by CaCO_3_ was 6 h, while to release it was 8 h. CNF was only able to adsorb 19% 5-FU for 24 h and release about 28.59%. Therefore, nanocellulose can be used to control the release of 5-FU on CaCO_3_, and the mixture and laminated models were used to carry out the total uptake and release process.

### 3.3. 3D Printed CaCO_3_-Nanocellulose Profiles

**Figure 4:**
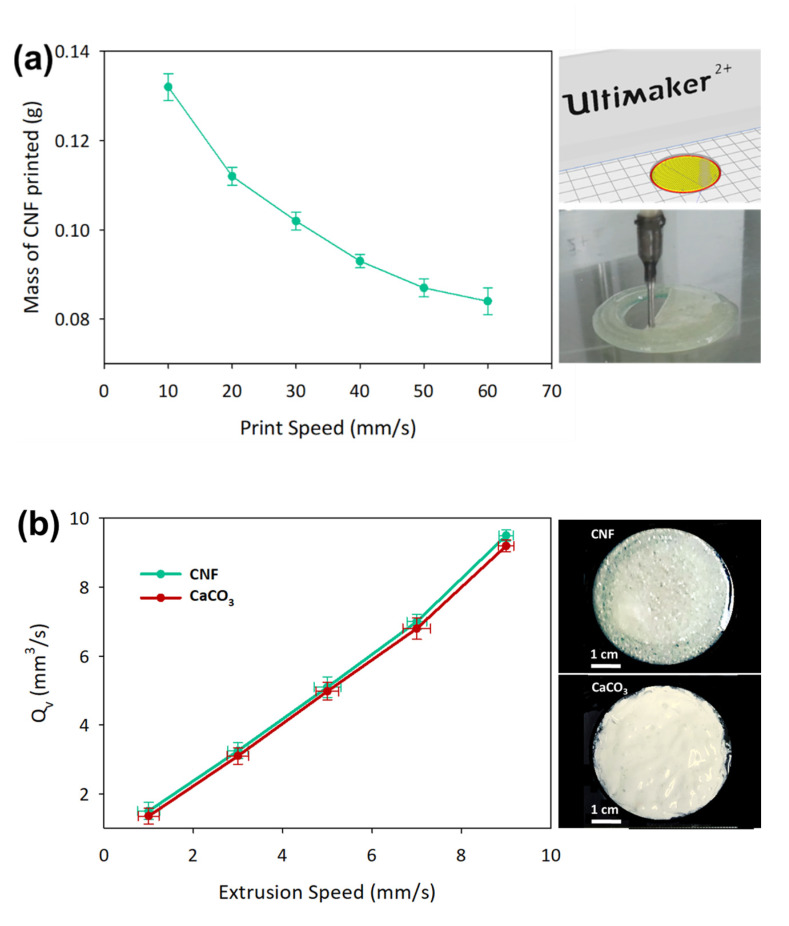

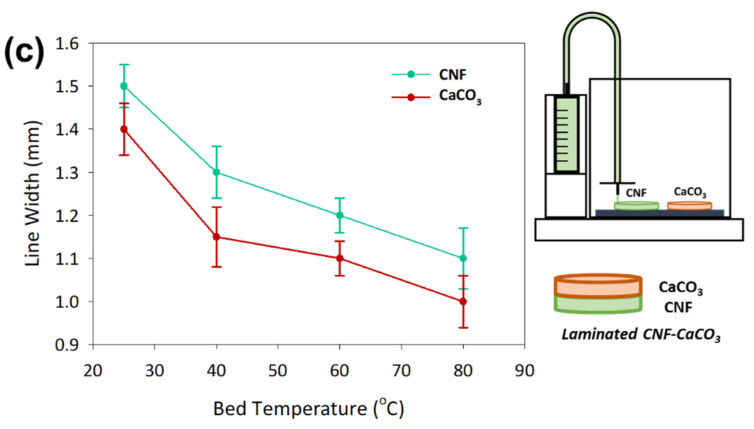
Parameters’ control in preparing 3D printed samples. (**a**) CNF at different printing speeds, (**b**) volumetric flow rates of CNF and CaCO_3_ and (**c**) the effect of build plate temperature on the printing line width. The data are presented as mean ± standard deviation (*n* = 3).

The initial investigation was done by a 3D printed circular model film with a dimension of 20 mm radius × 1.21 mm height at different print speeds. The actual mass of the printed CNF was determined after drying at 105 °C for 24 h, as shown in Figure 4a. Based on the theoretical calculation of the volume of the circular model and taking into account the moisture content, the total weight of 3D printed films is closely comparable with casted films, which have been fixed at 0.1 g dry weight basis. The printing profile was fixed at the print speed of 30 mm/s, which was able to attain 0.102 ± 0.014 g. Additionally, the printing parameters were improved by setting the line width at 1.21 mm, build plate temperature at 50 °C, extrusion speed at 3 mm/s, and print speed at 30 mm/s to attain a good printing profile. The volumetric flow rates of CNF and CaCO_3_, *Q*_v_, were measured with the variation of the extrusion speed of the syringe motor, as shown in Figure 4b. Both 7 wt % CNF and CaCO_3_ demonstrated similar flow rates at the same extrusion speed due to the shear-thinning properties of the liquid that assisted in the smooth extrusion process [43]. Extrusion speed of 3 mm/s was used for the subsequent printing experiments as the flow rate of solution could be controlled through the extrusion speed.

Further study was done on the effect of build plate temperature on the printing line width for CaCO_3_. A single line of 20 cm length was printed at 30 mm/s on different build plate temperatures to determine the optimum temperature to reduce smearing and evaporation issues. The drying of a thin film is known to cause issues such as contact line pinning, uneven surface tension, and a different pattern of pattern deposition [44]. When a high build plate temperature was used during the printing process, the CNF solution became gel-like due to the loss of water through the evaporation process. The line width printed on the build plate should follow the nozzle size used, which was 1.21 mm, and the temperature of 50 °C, as determined by referring to Figure 4c.

**Figure 5:**
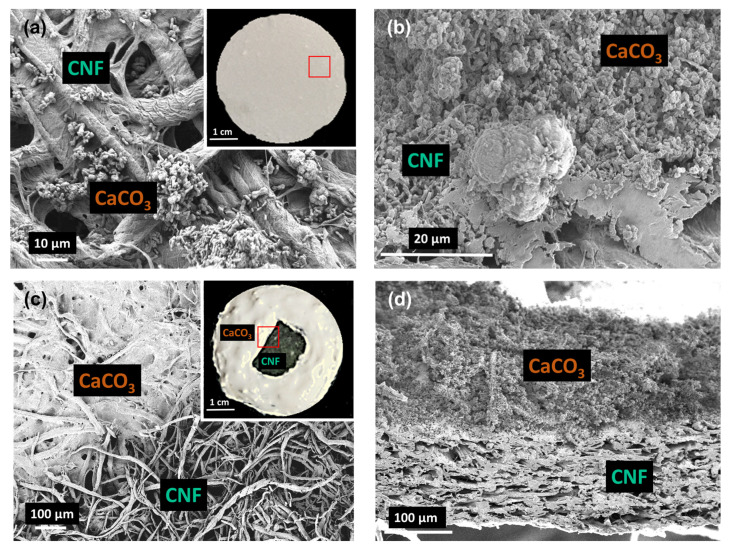
Morphological structure of the surface of (**a**) the CNF mixture with CaCO_3_, CaCO_3_–CNF–M (inset shows a 3D printed CaCO_3_–CNF–M), (**b**) the cross-sectional film of CaCO_3_–CNF–M, (**c**) CNF laminated with CaCO_3_, (inset shows a 3D printed CaCO_3_–CNF–L) and (**d**) the cross-sectional film of CaCO_3_–CNF–L.

Five different types of adsorption models were printed: CaCO_3_, CNF, CNF mixture with CaCO_3_ (CaCO_3_–CNF–M), CNF laminated with CaCO_3_ (CaCO_3_–CNF–L) and CNF double laminated with CaCO_3_ (CaCO_3_–CNF–DL). The printing profile was further supported by the morphological structure of the surface and cross-section of the 3D printed samples. As observed in Figure 5a, the CaCO_3_–CNF–M model shows a good dispersion of CaCO_3_ particles in the CNF matrix through both the surface and cross-section area. Figure 5b shows that the CaCO_3_ in CaCO_3_–CNF–L had been printed on the CNF matrix through the observation of the surface and cross-section and that obvious separation can be observed. The tensile strength of the films was evaluated to determine the influence of mixture and lamination in comparison to the CNF film in the 3D layer by layer deposition. The tensile strength of the mixture and laminated CaCO_3_ and CNF was evaluated at 63.1 ± 2.1 and 54.13 ± 1.9 MPa, respectively, whereas the CNF film shows higher mechanical properties of the tensile strength and Young’s modulus of 75.15 ± 2.3 and 733.51 ± 7.4 MPa.

### 3.4. Controlled-Release 5-FU

**Figure 6:**
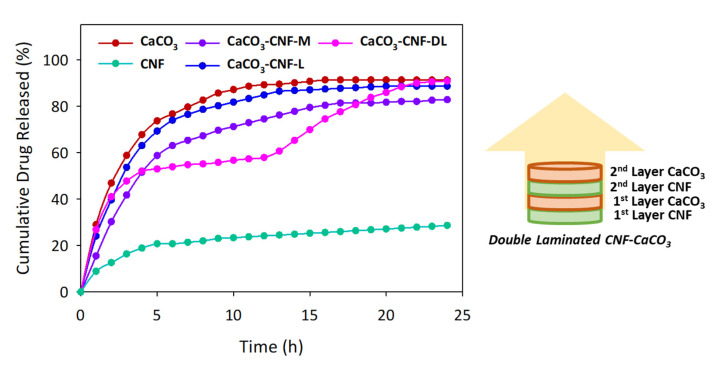
Kinetics release of 6 ppm of 5-FU using different 3D printed films of CNF, CaCO_3_, CNF mixture with CaCO_3_ (CaCO_3_–CNF–M), CNF laminated with CaCO_3_ (CaCO_3_–CNF–L) and CNF double laminated with CaCO_3_ (CaCO_3_–CNF–DL) for 24 h. The data are presented as mean (*n* = 3).

A controlled release of drug profile in 5-FU is beneficial as it minimizes the rapid initial release of drugs and the side effects due to the high toxicity towards cells by 5-FU [45]. The 5-FU release, which was adsorbed at 6 ppm on the selected medium was performed using PBS solution with a pH value of 7.4, which corresponded to the conditions of the small intestine fluid, for 24 h. A previous study performed showed a linear relationship of the Beer-Lambert law [46]. The controlled release profile was studied using one-way ANOVA (see Table S1, *p* < 0.05) for CaCO_3_, CNF, CNF mixture with CaCO_3_ (CaCO_3_–CNF–M), CNF laminated with CaCO_3_ (CaCO_3_–CNF–L) and CNF double laminated with CaCO_3_ (CaCO_3_–CNF–DL). The statistical analysis was simplified as shown in Table 2 and interpreted in Figure 6. The kinetics data observed at the 4th hour for CaCO_3_–CNF–M and CaCO_3_–CNF–DL have the same group due to the slowing down of the burst release, while CaCO_3_–CNF-DL has the same group as CaCO_3_–CNF–M and CaCO_3_–CNF–L at the 20th and 23^rd^ hour due to the staged drug release caused by the CNF layer covering the first layer of CaCO_3_ after the 13th hour.

As compared to the casted films of CNF, CaCO_3_ and CaCO_3_–CNF, shown previously in Table 1, the uptake and release behavior is significantly lower compared to the 3D printed version. The distinct adsorption-desorption phenomena between two techniques could be due to the random arrangement of the pores, whereby more pits are observed on the casted film and the pore structure are not in an orderly manner [47]. As shown in Figure 6, all the medium released performed the initial burst release within the first 2 h at 8.86% to 46.83% because 5-FU uptake was localized at the surface or the cross-section of the samples [48]. After the subsequent hours, the release of the drug decreased significantly for all samples except for CaCO_3_ as the nanocellulose lowered the surface area and controlled the diffusion process of the drug to the solution. CaCO_3_–CNF–L showed a higher release of 5-FU at 88.57% compared to CaCO_3_–CNF–M at 82.01% at equilibrium due to a higher surface area of exposed CaCO_3_, thus facilitating in a higher release of 5-FU compared to CaCO_3_–CNF–M. 

Although the release of the mixture of and single laminated CaCO_3_ and CNF was particularly lower, CaCO_3_–CNF–DL showed a significantly higher and staged release of 5-FU at the equilibrium point. 5-FU released from CaCO_3_–CNF–DL reached the first phase after the 4th hour and started to release for the second phase at the 13th hour until it reached the final equilibrium. The staged release was due to the CNF layer covering the first layer of CaCO_3_, preventing the burst of 5-FU release. The intraparticle diffusion mechanism of 5-FU between CaCO_3_ and CNF layers was taking place and initiating the second phase of 5-FU release. This is an ideal mechanism for a controlled drug delivery over time to avoid medication being taken regularly. Thus, the CaCO_3_–CNF–DL model can be used by utilizing CNF as the drug carrier and achieving the objective of the controlled release to allow it to last longer in the body system.

## 4. Conclusions

In this study, the integration of polymer composite was synthesized by successfully isolating and defibrillating cellulose from oil palm empty fruit bunch fibres and precipitating CaCO_3_ from the reaction of calcium chloride and sodium carbonate. CaCO_3_ has a porous structure and high surface area, making it an ideal drug carrier; thus, laminating them with nanocellulose could assist in the controlled release of the drugs loaded by physical embedment. Different models of CNF and CaCO_3_ were 3D printed and dried before the loading of 5-FU. Then, the drugs were released in a solution that mimicked the colon’s conditions. CaCO_3_–CNF–DL had a controlled release over 24 h due to the presence of nanocellulose that limited the drug release.

## Figures and Tables

**Table 1:** Kinetics data of the total uptake and the release at different initial concentrations of 5-FU on the casted films of CNF, CaCO_3_ and CaCO_3_–CNF. The data are presented as mean ± standard deviation (*n* = 3).

Samples	*C*_0_ (ppm)	*C*_e_ (ppm)	*q*_e_ (mg/g)	Uptake (%)	Release (%)
CaCO_3_	1	0.21	0.24	78.57 ± 0.34	80.52 ± 0.35
	2	0.42	0.47	79.10 ± 0.34	84.91 ± 0.18
	4	0.71	0.99	82.16 ± 0.41	87.77 ± 0.47
	6	1.01	1.50	83.19 ± 0.35	91.20 ± 0.24
	8	1.49	1.95	81.35 ± 0.41	86.92 ± 0.46
	10	1.90	2.43	80.96 ± 0.26	86.51 ± 0.42
CNF	1	0.84	0.05	15.12 ± 0.21	24.91 ± 0.18
	2	1.67	0.09	16.71 ± 0.19	26.12 ± 0.22
	4	3.25	0.23	18.12 ± 0.20	27.54 ± 0.23
	6	4.83	0.35	19.86 ± 0.26	28.59 ± 0.21
	8	6.30	0.51	21.37 ± 0.24	29.12 ± 0.27
	10	7.67	0.69	23.11 ± 0.17	30.86 ± 0.32
CaCO_3_–CNF	1	0.26	0.22	73.94 ± 0.37	77.42 ± 0.26
	2	0.47	0.46	76.45 ± 0.38	80.12 ± 0.34
	4	0.78	0.97	80.43 ± 0.36	83.38 ± 0.28
	6	1.09	1.47	81.83 ± 0.34	86.45 ± 0.37
	8	1.58	1.93	80.25 ± 0.40	82.67 ± 0.36
	10	1.99	2.40	80.12 ± 0.33	82.47 ± 0.35

*C*_0_ and *C*_e_, initial and equilibrium concentrations of the 5-FU (mg/L); *q*_e_, total uptake amount of 5-FU at final equilibrium.

**Table 2:** Kinetics data of 3D printed films of CNF, CaCO_3_, CNF mixture with CaCO_3_ (CaCO_3_–CNF–M), CNF laminated with CaCO_3_ (CaCO_3_–CNF–L) and CNF double laminated with CaCO_3_ (CaCO_3_–CNF–DL) for 24 h. The data are presented as mean ± standard deviation (*n* = 3).

Time (h)	5-FU Release (%)				
	CaCO_3_	CNF	CaCO_3_–CNF–M	CaCO_3_–CNF–L	CaCO_3_–CNF–DL
0	0.00 ± 0.00	0.00 ± 0.00	0.00 ± 0.00	0.00 ± 0.00	0.00 ± 0.00
1	28.94 ± 0.09 ^a^	8.86 ± 0.11 ^e^	15.44 ± 0.07 ^d^	24.04 ± 0.13 ^c^	26.80 ± 0.11 ^b^
2	46.83 ± 0.12 ^a^	12.61 ± 0.15 ^e^	30.20 ± 0.13 ^d^	39.62 ± 0.17 ^c^	40.96 ± 0.14 ^b^
3	58.75 ± 0.24 ^a^	16.35 ± 0.17 ^e^	41.68 ± 0.23 ^d^	53.65 ± 0.27 ^b^	47.73 ± 0.16 ^c^
4	67.70 ± 0.20 ^a^	18.85 ± 0.18 ^d^	51.51 ± 0.27 ^c^	63.00 ± 0.37 ^b^	52.04 ± 0.21 ^c^
5	73.66 ± 0.18 ^a^	20.72 ± 0.12 ^e^	58.73 ± 0.28 ^c^	69.24 ± 0.38 ^b^	52.96 ± 0.24 ^d^
6	76.64 ± 0.21 ^a^	20.72 ± 0.16 ^e^	62.99 ± 0.32 ^c^	73.92 ± 0.27 ^b^	53.88 ± 0.21 ^d^
7	79.62 ± 0.25 ^a^	21.35 ± 0.14 ^e^	65.28 ± 0.22 ^c^	76.41 ± 0.31 ^b^	54.80 ± 0.26 ^d^
8	82.60 ± 0.28 ^a^	21.97 ± 0.13 ^e^	67.25 ± 0.26 ^c^	78.59 ± 0.20 ^b^	55.11 ± 0.30 ^d^
9	85.58 ± 0.18 ^a^	22.97 ± 0.12 ^e^	69.55 ± 0.21 ^c^	80.15 ± 0.23 ^b^	55.73 ± 0.31 ^d^
10	87.07 ± 0.27 ^a^	23.35 ± 0.11 ^e^	71.19 ± 0.20 ^c^	81.71 ± 0.21 ^b^	56.65 ± 0.34 ^d^
11	88.56 ± 0.28 ^a^	23.72 ± 0.15 ^e^	72.83 ± 0.21 ^c^	83.27 ± 0.29 ^b^	57.27 ± 0.24 ^d^
12	89.16 ± 0.18 ^a^	24.09 ± 0.16 ^e^	74.47 ± 0.22 ^c^	84.83 ± 0.30 ^b^	57.88 ± 0.26 ^d^
13	89.46 ± 0.29 ^a^	24.47 ± 0.21 ^e^	76.11 ± 0.23 ^c^	86.39 ± 0.34 ^b^	60.65 ± 0.27 ^d^
14	90.05 ± 0.31 ^a^	24.84 ± 0.24 ^e^	77.75 ± 0.19 ^c^	86.70 ± 0.31 ^b^	65.27 ± 0.21 ^d^
15	90.65 ± 0.16 ^a^	25.22 ± 0.35 ^e^	79.38 ± 0.32 ^c^	87.01 ± 0.38 ^b^	69.88 ± 0.28 ^d^
16	91.19 ± 0.17 ^a^	25.59 ± 0.26 ^e^	80.37 ± 0.42 ^c^	87.32 ± 0.40 ^b^	74.50 ± 0.21 ^d^
17	91.21 ± 0.25 ^a^	25.97 ± 0.21 ^e^	81.35 ± 0.41 ^c^	87.63 ± 0.33 ^b^	77.58 ± 0.31 ^d^
18	91.21 ± 0.19 ^a^	26.09 ± 0.19 ^e^	81.37 ± 0.24 ^c^	87.75 ± 0.21 ^b^	78.45 ± 0.24 ^d^
19	91.22 ± 0.17 ^a^	26.21 ± 0.26 ^e^	81.39 ± 0.18 ^c^	87.83 ± 0.24 ^b^	79.11 ± 0.21 ^d^
20	91.22 ± 0.21 ^a^	26.34 ± 0.24 ^d^	81.39 ± 0.27 ^c^	87.94 ± 0.35 ^b^	80.65 ± 0.40 ^c^
21	91.26 ± 0.24 ^a^	26.72 ± 0.26 ^e^	81.46 ± 0.21 ^d^	88.26 ± 0.39 ^b^	83.73 ± 0.41 ^c^
22	91.33 ± 0.19 ^a^	27.09 ± 0.27 ^e^	81.68 ± 0.21 ^d^	88.57 ± 0.38 ^b^	85.88 ± 0.34 ^c^
23	91.35 ± 0.23 ^a^	27.47 ± 0.28 ^d^	82.01 ± 0.24 ^c^	88.61 ± 0.31 ^b^	88.35 ± 0.36 ^b^
24	91.37 ± 0.16 ^a^	27.84 ± 0.26 ^e^	82.06 ± 0.21 ^d^	88.63 ± 0.35 ^c^	89.88 ± 0.37 ^b^

For each group an ANOVA analysis was performed, with different letters indicating significant differences (*p* < 0.05).

## Supplementary Materials

The following are available online at https://www.mdpi.com/2073-4360/12/4/986/s1.

## References

[1] Holban A.M., Grumezescu A.M. (2016). Nanoarchitectonics for Smart Delivery and Drug Targeting.

[2] Li J., Mooney D.J. (2016). Designing hydrogels for controlled drug delivery. Nat. Rev. Mater..

[3] Li J., Wang X., Zhang T., Wang C., Huang Z., Luo X., Deng Y. (2015). A review on phospholipids and their main applications in drug delivery systems. Asian J. Pharm. Sci..

[4] Bruno B.J., Miller G.D., Lim C.S. (2013). Basics and recent advances in peptide and protein drug delivery. Ther. Deliv..

[5] Senapati S., Mahanta A.K., Kumar S., Maiti P. (2018). Controlled drug delivery vehicles for cancer treatment and their performance. Signal Transduct. Target Ther..

[6] Bozzuto G., Molinari A. (2015). Liposomes as nanomedical devices. Int. J. Nanomed..

[7] Zhang N., Yin Y., Xu S.J., Chen W.S. (2008). 5-Fluorouracil: Mechanisms of Resistance and Reversal Strategies. Molecules.

[8] Ortega-García M.B., Mesa A., Moya E.L.J., Rueda B., Lopez-Ordoño G., García J.Á., Conde V., Redondo-Cerezo E., Lopez-Hidalgo J.L., Jiménez G. (2020). Uncovering tumour heterogeneity through PKR and nc886 analysis in metastatic colon cancer patients treated with 5-FU-based chemotherapy. Cancers.

[9] Xu Z.Y., Tang J.N., Xie H.X., Du Y.A., Huang L., Yu P.F., Cheng X.D. (2015). 5-Fluorouracil Chemotherapy of Gastric Cancer Generates Residual Cells With Properties of Cancer Stem Cells. Int. J. Biol. Sci..

[10] Longley D.B., Harkin D.P., Johnston P.G. (2003). 5-fluorouracil: Mechanisms of action and clinical strategies. Nat. Rev. Cancer.

[11] Fischer F., Baerenfaller K., Jiricny J. (2007). 5-fluorouracil is efficiently removed from DNA by the base excision and mismatch repair systems. Gastroenterology.

[12] Chandran S.P., Natarajan S.B., Chandraseharan S., Suhaini M., Mohd B. (2017). Nano drug delivery strategy of 5-fluorouracil for the treatment of colorectal cancer. J. Cancer Res. Pract..

[13] Wigmore P.M., Mustafa S., El-Beltagy M., Lyons L., Umka J., Bennett G. (2010). Effects of 5-FU. Adv. Exp. Med. Biol..

[14] Thomas S.A., Grami Z., Mehta S., Patel K., North W., Hospital F. (2016). Adverse Effects of 5-fluorouracil: Focus on Rare Side Effects. Cancer Cell Microenviron..

[15] Tiwari G., Tiwari R., Sriwastawa B., Bhati L., Pandey S., Pandey P., Bannerjee S. (2012). Drug delivery systems: An updated review. Int. J. Pharm. Investig..

[16] O’Donnell K.L., Oporto-Velásquez G.S., Comolli N. (2020). Evaluation of Acetaminophen Release from Biodegradable Poly (Vinyl Alcohol) (PVA) and Nanocellulose Films Using a Multiphase Release Mechanism. Nanomaterials.

[17] Xu C., Molino B.Z., Wang X., Cheng F., Xu W., Molino P., Bacher M., Su D., Rosenau T., Willför S. (2018). 3D printing of nanocellulose hydrogel scaffolds with tunable mechanical strength towards wound healing application. J. Mater. Chem. B.

[18] Sultan S., Siqueira G., Zimmermann T., Mathew A.P. (2017). 3D printed of nano-cellulosic biomaterials for medical applications. Curr. Opin. Biomed. Eng..

[19] Leppiniemi J., Lahtinen P., Paajanen A., Mahlberg R., Metsä-Kortelainen S., Pinomaa T., Pajari H., Vikholm-Lundin I., Pursula P., Hytönen V.P. (2017). 3D-Printable Bioactivated Nanocellulose-Alginate Hydrogels. ACS Appl. Mater. Interfaces.

[20] Mariani L.M., Turner K.T., Iii W.R.J., Considine J.M. (2019). Printing and mechanical characterization of cellulose nanofibril materials. Cellulose.

[21] Kuzmenko V., Karabulut E., Pernevik E., Enoksson P., Gatenholm P. (2018). Tailor-made conductive inks from cellulose nanofibrils for 3D printing of neural guidelines. Carbohydr. Polym..

[22] Viidik L., Seera D., Antikainen O., Kogermann K., Heinämäki J., Laidmäe I. (2019). 3D-printability of aqueous poly(ethylene oxide) gels. Eur. Polym. J..

[23] Borujeni S.H., Mirdamadian S.Z., Varshosaz J., Taheri A. (2020). Three-dimensional (3D) printed tablets using ethyl cellulose and hydroxypropyl cellulose to achieve zero order sustained release profile. Cellulose.

[24] Auvinen V., Virtanen J., Merivaara A., Virtanen V., Laurén P., Tuukkanen S., Laaksonen T. (2020). Modulating sustained drug release from nanocellulose hydrogel by adjusting the inner geometry of implantable capsules. J. Drug Deliv. Sci. Technol..

[25] Islam K., Zuki B., Mustapha M., Mohd Z., Norshazlirah S., Eaqub A. (2011). Characterisation of calcium carbonate and its polymorphs from cockle Shells. Powder Technol..

[26] Maleki Dizaj S., Sharifi S., Ahmadian E., Eftekhari A., Adibkia K., Lotfipour F. (2019). An update on calcium carbonate nanoparticles as cancer drug/gene delivery system. Expert Opin. Drug Deliv..

[27] Pachuau L. (2017). Application of Nanocellulose for Controlled Drug Delivery.

[28] Sajab M.S., Mohan D., Santanaraj J., Chia C.H., Kaco H., Harun S., Kamarudin N.H.N. (2019). Telescopic synthesis of cellulose nanofibrils with a stable dispersion of Fe(0) nanoparticles for synergistic removal of 5-fluorouracil. Sci. Rep..

[29] Mohan D., Sajab M.S., Kaco H., Bakarudin S.B., Mohamed Noor A. (2019). 3D Printing of UV-Curable Polyurethane Incorporated with Surface-Grafted Nanocellulose. Nanomaterials.

[30] Santanaraj J., Sajab M.S., Mohammad A.W., Harun S., Chia C.H., Zakari S., Kaco H. (2017). Enhanced delignification of oil palm empty fruit bunch fibers with in situ fenton-oxidation. BioResources.

[31] Qurratu W.N., Wan Manan A., Santanaraja J., Sajab M.S., Wan Isahak W.N.R., Hua Chia C. (2018). Discoloration of Batik Effluent by Chemically Modified Oil Palm Empty Fruit Bunch Fibers. J. Kejuruter..

[32] Guo X., Liu L., Wang W., Zhang J., Wang Y., Yu S.-H. (2011). Controlled crystallization of hierarchical and porous calcium carbonate crystals using polypeptide type block copolymer as crystal growth modifier in a mixed solution. CrystEngComm.

[33] Salomão R., Costa L.M.M., Olyveira G.M. (2017). Precipitated calcium carbonate nano-microparticles: Applications in drug delivery. Adv. Tissue Eng. Regen. Med..

[34] Palamaea S., Dechatiwongse P., Choorita W., Chistid Y., Prasertsan P. (2017). Cellulose and hemicellulose recovery from oil palm empty fruit bunch (EFB) fibers and production of sugars from the fibers. Carbohydr. Polym..

[35] Chu D.H., Vinoba M., Bhagiyalakshmi M., Baek I.H., Nam S.C., Yoon Y., Kim S.H., Jeong S.K. (2013). CO_2_ mineralization into different polymorphs of CaCO_3_ using an aqueous-CO_2_ system. RSC Adv..

[36] Gong J., Li J., Xu J., Xiang Z., Mo L. (2017). Research on cellulose nanocrystals produced from cellulose sources with various polymorphs. RSC Adv..

[37] Hu Q., Zhang J., Teng H., Becker U. (2012). Growth process and crystallographic properties of ammonia-induced vaterite. Am. Mineral.

[38] Donnelly F.C., Purcell-Milton F., Framont V., Cleary O., Dunne P.W., Gun’ko Y.K. (2017). Synthesis of CaCO_3_ nano- and micro-particles by dry ice carbonation. Chem. Commun..

[39] Azali N.S., Kamarudin N.H.N., Rahim A.R.A., Nasir N.S.A.J., Timmiati S.N., Jaafar N.F. (2019). Adsorption and Release of 5-Fluorouracil (5FU) from Mesoporous Silica Nanoparticles. Mater. Today Proc..

[40] Sulekova M., Vahovska L., Hudak A., Zid L., Zelenak V. (2019). A Study of 5-fluorouracil desorption from mesoporous silica by RP-UHPLC. Molecules.

[41] Chong K.Y., Chia C.H., Zakaria S., Sajab M.S. (2014). Vaterite calcium carbonate for the adsorption of Congo red from aqueous solutions. J. Environ. Chem. Eng..

[42] Salimi S., Sotudeh-Gharebagh R., Zarghami R., Chan S.Y., Yuen K.H. (2019). Production of nanocellulose and its applications in drug delivery: A critical review. ACS Sustain. Chem. Eng..

[43] Quennouz N., Hashmi S.M., Choi H.S., Kim J.W., Osuji C.O. (2016). Rheology of cellulose nanofibrils in the presence of surfactants. Soft Matter..

[44] Okuzono T., Kobayashi M., Doi M. (2009). Final shape of a drying thin film. Phys. Rev. E.

[45] Dalei G., Swain S., Das S., Das S.P. (2020). Controlled Release of 5-Fluorouracil from Alginate Hydrogels by Cold HMDSO-Plasma Surface Engineering. ChemistrySelect.

[46] Ibrahim R.A.A., Shareef F., Suhail A., Al-hakeim H.K. (2018). Stability of Anticancer Drug 5-Fluorouracil in Aqueous Solution: An Assessment of Kinetic Behavior. Nano Biomed. Eng..

[47] Zhang H.Y., Jiang H.B., Ryu J.H., Kang H., Kim K.M., Kwon J.S. (2019). Comparing properties of variable pore-sized 3D-printed PLA membrane with conventional PLA membrane for guided bone/tissue regeneration. Materials.

[48] Priya P., Raja A., Raj V. (2016). Interpenetrating polymeric networks of chitosan and egg white with dual crosslinking agents polyethylene glycol/polyvinylpyrrolidone as a novel drug carrier. Cellulose.

