# Supplementary material for: 3D Printed Laminated CaCO3-Nanocellulose Films as Controlled-Release 5-Fluorouracil
Source: Polymers (Basel). 2020 Apr 23;12(4):986. doi: 10.3390/polym12040986 (PMC7240736; doi:10.3390/polym12040986)
Supplement: Supplementary file 1 [file polymers-12-00986-s001.pdf]

3D Printed Laminated $CaCO_3$-nanocellulose Films as Controlled-release 5-fluorouracil

Denesh Mohan, Nur Fatin Khairullah, Yan Ping How, Mohd Shaiful Sajab and Hatika Kaco

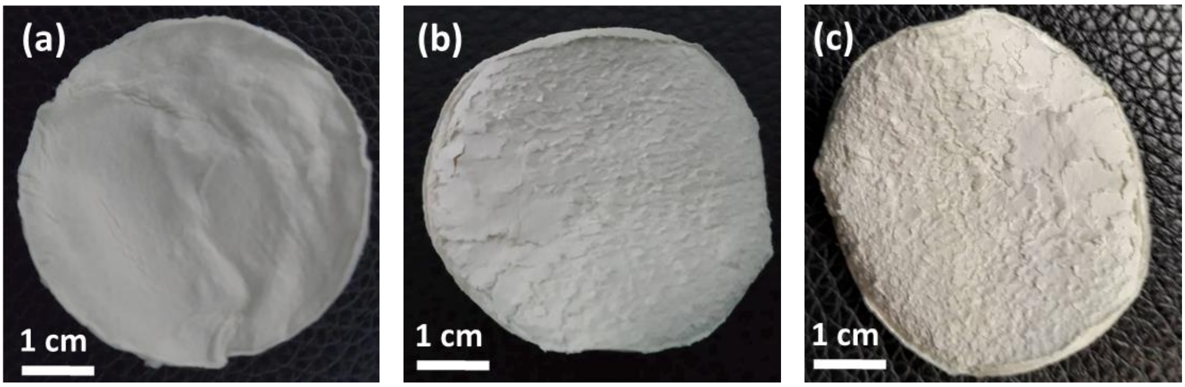

Figure S1. Casted films of (**a**) CNF, (**b**) $CaCO_3$ and (**c**) $CaCO_3$-CNF on the top of nylon membrane filter (47 mm diameter, 0.45 μm pore size) using vacuum filtration technique.

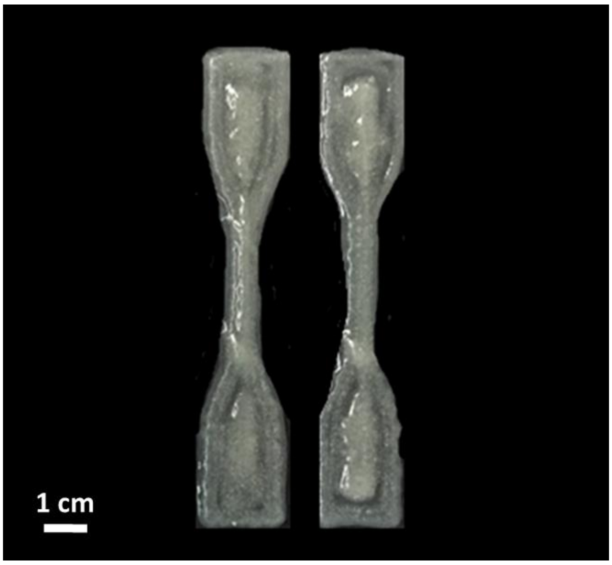

Figure S2. Liquid deposition modelling of 7 wt % CNF suspension followed by a standard tensile specimen 3D model according to the ASTM D638 Type IV (printing and slicing profiles; nozzle diameter: 1.21 mm; printing speed: 30 mm/s; extrusion speed: 3 mm/s; build plate temperature: 50 °C).

Table S1. One-way ANOVA results on kinetic release of 5-FU (%) for 24 h (Tukey Method; N: 3; α = 0.05; $p \leq 0.05$; Equal variances were assumed for the analysis)

Samples	Time (h)	Mean	StDev	95% CI	Grouping
$CaCO_3$		28.943	0.087	(28.810, 29.076)	A
$CaCO_3$-CNF-DL		26.800	0.108	(26.666, 26.931)	B
$CaCO_3$-CNF-L	1	24.043	0.131	(23.910, 24.176)	C
$CaCO_3$-CNF-M		15.437	0.070	(15.303, 15.569)	D
CNF		8.857	0.111	(8.723, 8.989)	E
$CaCO_3$		46.827	0.123	(46.644, 47.009)	A
$CaCO_3$-CNF-DL		40.963	0.140	(40.781, 41.146)	B
$CaCO_3$-CNF-L	2	39.620	0.170	(39.437, 39.803)	C
$CaCO_3$-CNF-M		30.200	0.125	(30.017, 30.383)	D
CNF		12.613	0.146	(12.431, 12.796)	E
$CaCO_3$		58.750	0.241	(58.472, 59.028)	A
$CaCO_3$-CNF-DL		47.727	0.158	(47.441, 48.005)	C
$CaCO_3$-CNF-L	3	53.647	0.267	(53.368, 53.925)	B
$CaCO_3$-CNF-M		41.677	0.229	(41.398, 41.955)	D
CNF		16.350	0.165	(16.072, 16.628)	E
$CaCO_3$		67.703	0.200	(67.374, 68.033)	A
$CaCO_3$-CNF-DL		52.043	0.212	(51.714, 52.373)	C
$CaCO_3$-CNF-L	4	63.003	0.366	(62.674, 63.333)	B
$CaCO_3$-CNF-M		51.513	0.274	(51.184, 51.843)	C
CNF		18.847	0.182	(18.517, 19.176)	D
$CaCO_3$		73.657	0.180	(73.328, 73.985)	A
$CaCO_3$-CNF-DL		52.957	0.240	(52.628, 53.285)	D
$CaCO_3$-CNF-L	5	69.237	0.377	(68.908, 69.565)	B
$CaCO_3$-CNF-M		58.727	0.284	(58.398, 59.055)	C
CNF		20.723	0.117	(20.394, 21.052)	E
$CaCO_3$		76.643	0.206	(76.335, 76.952)	A
$CaCO_3$-CNF-DL		53.880	0.207	(53.572, 54.188)	D
$CaCO_3$-CNF-L	6	73.920	0.272	(73.612, 74.228)	B
$CaCO_3$-CNF-M		62.987	0.322	(62.678, 63.295)	C
CNF		20.723	0.157	(20.415, 21.032)	E
$CaCO_3$		79.620	0.250	(79.306, 79.934)	A
$CaCO_3$-CNF-DL		54.803	0.263	(54.490, 55.117)	D
$CaCO_3$-CNF-L	7	76.407	0.310	(76.093, 76.720)	B
$CaCO_3$-CNF-M		65.283	0.220	(64.970, 65.597)	C
CNF		21.350	0.144	(21.037, 21.664)	E

$CaCO_3$		82.603	0.276	(82.292, 82.915)	A
$CaCO_3$-CNF-DL		55.110	0.303	(54.799, 55.421)	D
$CaCO_3$-CNF-L	8	78.587	0.203	(78.275, 78.898)	B
$CaCO_3$-CNF-M		67.247	0.260	(66.935, 67.558)	C
CNF		21.967	0.129	(21.655, 22.278)	E
$CaCO_3$		85.583	0.180	(85.303, 85.864)	A
$CaCO_3$-CNF-DL		55.727	0.305	(55.446, 56.007)	D
$CaCO_3$-CNF-L	9	80.153	0.230	(79.873, 80.434)	B
$CaCO_3$-CNF-M		69.550	0.211	(69.269, 69.831)	C
CNF		22.973	0.121	(22.694, 23.254)	E
$CaCO_3$		87.070	0.266	(86.766, 87.374)	A
$CaCO_3$-CNF-DL		56.650	0.340	(56.346, 56.954)	D
$CaCO_3$-CNF-L	10	81.707	0.206	(81.403, 82.011)	B
$CaCO_3$-CNF-M		71.193	0.195	(70.889, 71.497)	C
CNF		23.347	0.111	(23.045, 23.651)	E
$CaCO_3$		88.560	0.282	(88.251, 88.869)	A
$CaCO_3$-CNF-DL		57.267	0.240	(56.958, 57.575)	D
$CaCO_3$-CNF-L	11	83.270	0.295	(82.961, 83.579)	B
$CaCO_3$-CNF-M		72.833	0.206	(72.525, 73.142)	C
CNF		23.717	0.146	(23.408, 24.025)	E
$CaCO_3$		89.160	0.175	(88.863, 89.457)	A
$CaCO_3$-CNF-DL		57.877	0.261	(57.580, 58.173)	D
$CaCO_3$-CNF-L	12	84.830	0.302	(84.533, 85.127)	B
$CaCO_3$-CNF-M		74.467	0.221	(74.170, 74.763)	C
CNF		24.093	0.164	(23.797, 24.390)	E
$CaCO_3$		89.457	0.293	(89.106, 89.807)	A
$CaCO_3$-CNF-DL		60.650	0.270	(60.300, 61.000)	D
$CaCO_3$-CNF-L	13	86.387	0.340	(86.036, 86.737)	B
$CaCO_3$-CNF-M		76.107	0.230	(75.756, 76.457)	C
CNF		24.473	0.207	(24.123, 24.824)	E
$CaCO_3$		90.050	0.312	(89.721, 90.379)	A
$CaCO_3$-CNF-DL		65.270	0.205	(64.941, 65.599)	D
$CaCO_3$-CNF-L	14	86.700	0.308	(86.371, 87.029)	B
$CaCO_3$-CNF-M		77.747	0.191	(77.418, 78.075)	C
CNF		24.843	0.236	(24.515, 25.172)	E

$CaCO_3$	15	90.650	0.160	(90.254, 91.045)	A
$CaCO_3$-CNF-DL		69.880	0.278	(69.484, 70.276)	D
$CaCO_3$-CNF-L		87.013	0.382	(86.617, 87.409)	B
$CaCO_3$-CNF-M		79.380	0.320	(78.984, 79.776)	C
CNF		25.217	0.349	(24.821, 25.613)	E
$CaCO_3$	16	91.193	0.168	(90.795, 91.590)	A
$CaCO_3$-CNF-DL		74.500	0.214	(74.103, 74.897)	D
$CaCO_3$-CNF-L		87.323	0.398	(86.926, 87.721)	B
$CaCO_3$-CNF-M		80.373	0.423	(79.976, 80.771)	C
CNF		25.593	0.257	(25.196, 25.991)	E
$CaCO_3$	17	91.193	0.168	(90.796, 91.591)	A
$CaCO_3$-CNF-DL		74.500	0.214	(74.103, 74.897)	D
$CaCO_3$-CNF-L		87.323	0.398	(86.926, 87.721)	B
$CaCO_3$-CNF-M		80.373	0.423	(79.976, 80.771)	C
CNF		25.593	0.257	(25.196, 25.991)	E
$CaCO_3$	18	91.207	0.182	(90.933, 91.480)	A
$CaCO_3$-CNF-DL		78.447	0.236	(78.173, 78.720)	D
$CaCO_3$-CNF-L		87.753	0.208	(87.480, 88.027)	B
$CaCO_3$-CNF-M		81.370	0.236	(81.097, 81.643)	C
CNF		26.090	0.193	(25.817, 26.363)	E
$CaCO_3$	19	91.217	0.170	(90.939, 91.494)	A
$CaCO_3$-CNF-DL		79.117	0.212	(78.839, 79.394)	D
$CaCO_3$-CNF-L		87.837	0.238	(87.559, 88.114)	B
$CaCO_3$-CNF-M		81.390	0.183	(81.112, 81.668)	C
CNF		26.213	0.263	(25.936, 26.491)	E
$CaCO_3$	20	91.223	0.215	(90.832, 91.615)	A
$CaCO_3$-CNF-DL		80.653	0.404	(80.262, 81.045)	C
$CaCO_3$-CNF-L		87.937	0.348	(87.545, 88.328)	B
$CaCO_3$-CNF-M		81.393	0.274	(81.002, 81.785)	C
CNF		26.340	0.238	(25.949, 26.731)	D
$CaCO_3$	21	91.263	0.240	(90.861, 91.665)	A
$CaCO_3$-CNF-DL		83.733	0.406	(83.331, 84.135)	C
$CaCO_3$-CNF-L		88.257	0.392	(87.855, 88.659)	B
$CaCO_3$-CNF-M		81.457	0.211	(81.055, 81.859)	D
CNF		26.717	0.260	(26.315, 27.119)	E

$CaCO_3$	22	91.330	0.185	(90.960, 91.700)	A
$CaCO_3$-CNF-DL		85.877	0.342	(85.507, 86.246)	C
$CaCO_3$-CNF-L		88.573	0.382	(88.204, 88.943)	B
$CaCO_3$-CNF-M		81.677	0.214	(81.307, 82.046)	D
CNF		27.093	0.265	(26.724, 27.463)	E
$CaCO_3$	23	91.353	0.227	(90.982, 91.724)	A
$CaCO_3$-CNF-DL		88.350	0.356	(87.979, 88.721)	B
$CaCO_3$-CNF-L		88.610	0.314	(88.239, 88.981)	B
$CaCO_3$-CNF-M		82.010	0.241	(81.639, 82.381)	C
CNF		27.473	0.284	(27.102, 27.844)	D
$CaCO_3$	24	91.367	0.157	(91.006, 91.727)	A
$CaCO_3$-CNF-DL		89.877	0.370	(89.516, 90.237)	B
$CaCO_3$-CNF-L		88.630	0.348	(88.269, 88.991)	C
$CaCO_3$-CNF-M		82.060	0.210	(81.699, 82.421)	D
CNF		27.837	0.257	(27.476, 28.197)	E

Means that do not share a letter are significantly different; $p < 0.05$
